# Budget impact analysis of the adoption of new hypertension guidelines in Colombia

**DOI:** 10.1186/s12962-018-0152-5

**Published:** 2018-09-25

**Authors:** Cesar Augusto Guevara-Cuellar, Victoria Eugenia Soto, María Isabel Molina-Echeverry

**Affiliations:** 1 Facultad de Ciencias de la Salud, Universidad Icesi, Calle 18 #122-135, Cali, Valle del Cauca Colombia; 2 PROESA, Universidad Icesi, Calle 18 #122-135, Cali, Valle del Cauca Colombia

**Keywords:** Hypertension, Cardiovascular event, Practice guideline, Budget impact, Health care costs, American Heart Association

## Abstract

**Background:**

Hypertension represents a high burden of disease in different healthcare systems. Recent guideline published in 2017 by the American Heart Association and the American College of Cardiology has generated a debate between clinicians and policymakers due to the lowering of diagnosis threshold and the subsequent increase of the prevalence and healthcare costs. No empirical research exists addressing the question about the pressure on healthcare costs generated by new standards. This study aims to quantify the impact on the hypertension diagnosis and treatment costs for healthcare system using the new hypertension guideline.

**Methods:**

We conducted a budget impact analysis from a Colombian healthcare payer’s perspective with a 3-year time horizon (2018–2020), in which we estimated the difference in total medical care costs between previous hypertension cut-off points (140/90 mmHg) and new guideline cut-off points (130/80 mmHg).

**Results:**

Our results show that the impact of the adoption of the new hypertension guideline would represent a decrease close to 22% in total annual high blood pressure costs in Colombia. This reduction is mainly driven by a lower number of cardiovascular complications. It is worth noting that these results should be taken with caution due to local available data.

**Conclusions:**

A high-middle income country such as Colombia should carry out an exhaustive revision of the recommendations of the new hypertension guideline, due to its high probability of saving medical treatment costs for the healthcare system.

**Electronic supplementary material:**

The online version of this article (10.1186/s12962-018-0152-5) contains supplementary material, which is available to authorized users.

## Background

Hypertension represents a high burden of disease in different healthcare systems. The World Health Organization (WHO) attributes to hypertension at least 45% of deaths by cardiomyopathies, 51% of deaths by cerebrovascular diseases and costs near 1.26 billion dollars in high-middle income countries [1]. A recent report quantified the hypertension costs for healthcare systems of Latin America, including Colombia, by USD 999 and USD 199 million respectively in 2015 [2].

Different clinical practice guidelines about hypertension have shown a tendency to make a timely diagnosis and strict control of the disease given the evidence of the impact that these strategies can have on morbidity and mortality. The recent guideline published in 2017 by the American Heart Association (AHA) and the American College of Cardiology (ACC) is not far from this tendency [3]. This guideline for the detection, prevention, management and treatment of high blood pressure (HBP) redefined the cut-off points for systolic and diastolic blood pressure, with hypertension now defined as blood pressure higher than 130/80 mmHg rather than 140/90 mmHg.

However, it is estimated that lowering threshold to diagnose hypertension will raise the HBP prevalence by 46% and 50% which has generated considerable debate among clinicians and policymakers. The formers consider that the implementation of the new cut-off points for hypertension will make even harder to accomplish the treatment goals proposed by previous guidelines [4–6]. Furthermore, it is unknown the effect of the new guideline on the already overloaded function of primary care physicians [4–6]. There is a tendency to prescribe antihypertensive medication to the diagnosed hypertensive patients despite that management should be based on lifestyle changes [6, 7]. Thus, the new cut-off points for hypertension may lead to a higher rate of adverse effects derived from the treatment of newly diagnosed patients.

Regarding the healthcare system perspective, the adoption of the new guideline may come along with higher hypertension diagnosis costs. The definition of stricter target values to the hypertension management would also have an impact on costs due to higher-intensity treatment [6, 7]. Nonetheless, supporters of the new guideline sustain that lower cut-off points to diagnose hypertension account for cardiovascular complications that can occur later and hence, allow for earlier treatment intervention [3].

According to the AHA/ACC’s guideline authors, there would not be a substantial increase in annual medical treatment costs since only a small proportion (between 2 and 5%) of the patients diagnosed would require pharmacological treatment [3]. However, to our knowledge, no empirical research exists addressing the question about the economic pressure on healthcare system generated by the adoption of the new hypertension guideline.

This study aims to quantify the potential impact on the hypertension treatment costs for Colombian healthcare system by adopting the new cut-off points proposed by the new AHA/ACC’s guideline to both diagnosis and management of hypertension. Colombia is an interesting case of study due to the increasing prevalence of hypertension (5.5% in 2011 to 7.2% in 2015), and the low treatment rates, despite the enormous efforts to improve hypertension management in a context of financial healthcare system challenges [1]. This disease group accounts for about 6.6% of total healthcare expenditure in Colombia [2]. In addition, it accounts for a large percentage of non-fatal morbidity involving a 0.3% of national gross domestic product (GDP) because of sickness and disability [2].

## Methods

The budget impact model estimated the total medical care cost of adopting the new hypertension guideline based on the size of eligible patient population and the monotherapy treatment costs. Two hypothetical scenarios were defined according to cut-off points for hypertension diagnosis and treatment goals. The baseline scenario used the values of systolic or diastolic blood pressure higher than or equal to 140 or 90 mmHg respectively, as a cut-off point for hypertension diagnosis and, systolic blood pressure less than 140 mmHg as a treatment goal. The new scenario considered diagnosing hypertension with systolic or diastolic blood pressure values higher than or equal to 130 or 80 mmHg respectively and, systolic blood pressure less than 120 mmHg as a treatment goal. The potential budget impact was defined as the difference in total medical care costs between those two scenarios.

Regarding clinical and cost data of diagnosis and treatment of hypertension, local and international databases (i.e. EMBASE, PUBMED, LILACS, IETS, Ministry of Health and Social Protection, AHA) were consulted for descriptive local studies to find data on complications and costs. The number of events was searched in the SISPRO (The Integrated Information System of Social Protection of Colombia).

The analysis was conducted from a Colombian healthcare payer’s perspective with a 3-year time horizon (2018–2020), which is a relevant time horizon for the budget estimations according to local and international budgeting process and standards [8, 9]. In addition, 3-year time horizon allows the model to analyse the differences between different hypertension treatment goal effects and consequences in terms of costs associated with the adoption of the new guideline [10].

Improvements in quality of life or changes in clinical variables were not included in the analysis. We also assume that the fact of doctors having stricter hypertension treatment goals will translate into a greater survival rate. Therefore, in our model, the hypertension prevalence will increase in the following year according to both estimated prevalence due to the adoption of new standards and surviving population resulted by early treatment of cardiovascular complications. The model was estimated in Excel 2016 (Microsoft Corporation, Redmond, WA, USA) with the SimulAr simulation package.

### Population

The estimated population over 20 years old from the National Administrative Department of Statistics of Colombia (DANE) was used to determine the population for years 2018–2020. In baseline scenario, the number of patients with arterial hypertension was calculated according to the criteria of previous guidelines. The HBP prevalence under those parameters was 7.2% [1].

Given the limited availability of local data, the hypertension prevalence with the new cut-off points was taken from the data provided in the AHA’s clinical guideline [3]. We extrapolated the proportions of hypertensive patients in each age-group described in AHA’s guideline to Colombian population. This assumption might suppose a scenario with a relative higher proportion of hypertensive patients for Colombia context, although the AHA/ACC’s guideline is the only clinical study published so far providing HBP prevalence data on the potential effect of adopting the new treatment goals. As a result, in 2018, the hypertensive population in the baseline scenario is 8.675.154 and under the new scenario is 13.271.577 which represents an increase of 52%.

After the HBP prevalence was estimated, the proportion of patients who underwent diagnostic tests and received medication for each scenario were calculated taking into account the ratio of physicians adhering to guideline for hypertension management and the ratio of patients adhering to medical recommendations. The parameters used are presented in Table 1.

**Table 1:** Model parameters

Parameter	Estimation	Probabilistic sensitivity analysis		References
		Probability distribution	Distribution parameters	
Physician’s prescription probability	0.52	β	α: 29 β: 31	[18]
Patient’s probability of adherence to the medical recommendation (with BP > 140/90)	0.45	β	α: 45 β: 55	[19]
Patient’s probability of adherence (with SBP: 130–139 or DBP: 80–89 mmHg)	0.2	β	α: 20 β: 80	[35]
Annual probability of AMI in standard control (BP < 140/90 mmHg)	0.0078	β	α: 116 β: 4562	[11]
Annual probability of stroke in standard control (BP < 140/90 mmHg)	0.0047	β	α: 140 β: 4543	[11]
Annual probability of HF in standard control (BP < 140/90 mmHg)	0.0067	β	α: 100 β: 4583	[11]
Annual probability of death due to cardiovascular disease in standard control (BP < 140/90 mmHg)	0.0043	β	α: 65 β: 4618	[11]
Annual probability of AMI in intensive control (BP < 120/80 mmHg)	0.0065	β	α: 97 β: 4586	[11]
Annual probability of stroke in intensive control (BP < 120/80 mmHg)	0.0041	β	α: 62 β: 4616	[11]
Annual probability of HF in intensive control (BP < 120/80 mmHg)	0.0041	β	α: 62 β: 4616	[11]
Annual probability of death due to cardiovascular disease in intensive control (BP < 120/80 mmHg)	0.0025	β	α: 37 β: 4641	[11]
Annual probability of AMI in untreated patients^a^	0.047	β	α: 47 β: 953	[36]
Annual probability of stroke in untreated patients^a^	0.1040	β	α: 104 β: 896	[36]
Annual probability of HF in untreated patients^a^	0.0396	β	α: 39 β: 961	[36]
Mean of daily tablets in patients with intensive control (BP < 120/80 mmHg)	2.8	Poisson	λ: 2.8	[11]
Mean of daily tablets in patients with standard control (BP < 140/90 mmHg)	1.8	Poisson	λ: 1.8	[11]
Mean of annual prescription of diagnostic aids	0.61	Poisson	λ: 1.5	[37]
Mean of annual episodes decompensation of HF	2.2	Poisson	λ: 2	[38]
Weighted average daily cost of antihypertensive medication	0.00556	–	–	[39]
Average total cost of diagnostic aids	57.91	–	–	[40]
Average total cost per AMI per episode in 2018^b,c^	USD 2938	–	–	[21]
Average total cost per AMI per episode in 2019	USD 3235	–	–	[21]
Average total cost per AMI per episode in 2020	USD 2934	–	–	[21]
Average total cost of stroke^c^	USD 3430	γ	Alfa: 0,44604 β: 19360084	[22]
Average total cost per episode of decompensated HF^d^	USD 1990	γ	Alfa: 0,44248 β: 14526853	[23]
Average total cost of HF chronic management^e^	USD 1131	γ	Alfa: 0,1599 β: 1902392	[23]

^a^Data calibrated from the source

^b^Not modeled as a probability distribution due to absence of variance data

^c^Costs derived from acute episode care

^d^Costs derived from hospital care for an episode of decompensated HF

^e^Costs derived from outpatient care of patients with chronic HF

Subsequently, the proportion of patients presenting major cardiovascular events [Acute Myocardial Infarction (AMI), stroke, and heart failure (HF)] for each scenario was obtained from SPRINT study [11]. Notice here that patients in SPRINT study were at least 50 years-old and in contrast, our study included a younger population (younger than 50 years-old). However, we decided to use a population over-20 years old because observational studies have showed an increasing incidence of hypertension at younger ages. In an analysis of 1.132 white male medical students (mean age: close to 23 years at baseline) found that 0.3%, 6.5%, and 37% of the students developed hypertension at age 25, 45, and 65 years, respectively [12]. Other studies in Colombian students older than 18 years have found prevalence of high blood pressure oscillating between 12 and 43% [13, 14]. In the same line, other studies in the other countries have obtained similar conclusions [15–17]. Thus, we try to capture the potential increase of hypertension in younger population and the budgetary impact of this phenomenon under the adoption of new hypertension diagnosis standards.

**Fig. 1:**
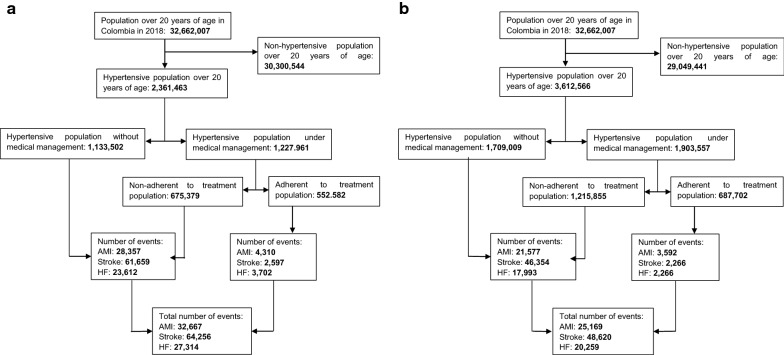
Estimated population by scenario, according to hypertension and cardiovascular events prevalence. Year 2018. **a** Baseline scenario. **b** New scenario

We assumed that population that survives each year is equal to those individuals who were diagnosed as hypertensive with the new cut-off points and followed physician prescriptions accordingly with medical guide recommendations (i.e. 0.52 and 0.2, respectively) [18, 19]. Figure 1 shows the estimated population by scenario in 2018. The same procedure was carried out to estimate the HBP prevalence in 2019 and 2020.

### Diagnosis and treatment costs

For each scenario, we calculated total average treatment costs that included medications, diagnostic aids and the acute and chronic management of complications (AMI, HF and Stroke) in patients of the target population. Costs for rehabilitation programs and outpatient treatment and follow-up were not included.

Blood sugar, complete blood count, creatinine, urinalysis, conventional electrocardiogram, electrolytes, lipid profile, serum calcium and, thyroid stimulating hormone were included as diagnostic aids.

Regarding medication costs, the most frequently prescribed antihypertensive drugs in Colombia were identified. Machado et al. [20] found that Losartan, Hydrochlorothiazide, Enalapril, and Metoprolol represent the four most frequent drugs prescribed in 20 cities of Colombia in 2013.

The Drug Price Information System of Colombia (SISMED) along with the average of the frequency of prescription were used to estimate the average daily cost of drugs, for the period of July–September of 2017. Regarding prescription, the values provided by the SPRINT study were taken into account: 2.8 daily medications were estimated for the intensive control scenario and 1.8 daily medications for the standard control [11].

Furthermore, the cost per episode and the cardiovascular complication costs associated with outpatient management (i.e. medications and non-surgical procedures) were taken from local studies (see Table 1).

The study aimed to quantify the average medical costs of myocardial infarction was performed in 213 patients admitted to a university hospital in Bogotá, Colombia with suspected acute coronary syndrome in 2010. In this study, the perspective of the provider was considered. This study found an average total cost of USD 2934 [21].

The cost study in stroke was performed in 166 patients older than 50 years who were admitted to a university hospital in Bogotá, Colombia between 2010 and 2013 with clinical stroke associated or not to atrial fibrillation. A perspective from the provider was adopted. This study found an average total cost USD 3430 [22].

The cost for heart failure was obtained from a study performed in two hospitals in Bogota in the 2011 which included 158 patients with an average age of 62 years and diagnosis of heart failure. The study was conducted from the perspective of the payer and included direct costs of outpatient and hospital treatment. Costs was estimated per episode of decompensated heart failure in 1990 USD to cost average total of outpatient management of USD 1131 [23].

Cost per cardiovascular episode were estimated in real values (i.e. deflated) by using Colombian Consumer Price Index (GDP equal to 3.95 in 2018, 4.35 in 2019 and 3.95 in 2020) [24]. Costs are reported in US dollars (1 USD = COP $2877; 22 February 2018). All parameters and model inputs are shown and referenced in Table 1.

### Validation and calibration of the model

Two clinicians from the investigation group conducted the validity face; they evaluated the structure, outcomes and possible treatment routes for patients in each scenario. The model’s internal validity was evaluated by performing univariate sensitivity analysis, black box test and running the model using extreme values [25, 26]. These common tests used in model validation helped us to determine whether the model behaved as intended (i.e. number of cardiovascular episodes) and had been implemented correctly. The model’s internal validity was evaluated by performing exploratory testing such as extreme values [23, 24]. These common tests used in model validation helped us to determine whether the model behaved as intended (i.e. number of cardiovascular episodes), to verify the internal mathematical logic and it had been implemented correctly. For further details on these techniques see Dasbach et al. [25].

The outputs of the model correspond to the total number of major cardiovascular events and total medical costs for both scenarios. For external validation, the model’s ability to quantify the number of events was evaluated, and contrasted with the number of benefits reported by the SISPRO for years 2018–2020. Events were defined according to ICD-10 codes as I219- Acute Myocardial Infarction, unspecified; I509- heart failure, unspecified and, I64- stroke, not specified as hemorrhage or infarction.

The estimated number of events with their corresponding 95% confidence interval was predicted by using a simple exponential smoothing method (alpha: 0.1) [27]. If the estimated number fell within the interval, it was considered that the model predicted adequately. Finally, the validation of the effect size of intensive versus standard treatment was performed by calculating the hazard ratio of the expected events for both scenarios. The estimated hazard ratios for AMI, stroke, and HF were equal to 0.86, 0.85 and, 0.82 respectively. These ratios were validated with the confidence intervals reported by the SPRINT study.

Confidence intervals for estimated cardiovascular events are available in Additional file 1.

### Sensitivity analysis

A probabilistic sensitivity analysis was performed by second-order Monte Carlo simulation with 5000 iterations. Table 1 also shows the probability distributions and their respective parameters.

The probability distribution Beta was used to modeling probabilities. In this distribution, alpha values represent the number of patients who presented the event of interest. Beta values represent the number of patients who did not show the event of interest. For modeling quantity of resources and number of episodes, a Poisson probability distribution was used. In this distribution, the lambda parameter represents the average number of events of interest. For modelling costs, we used a Gamma distribution, Beta parameter is the variance divided by expected value and alpha parameter is expected value divided by beta parameter.

## Results

**Fig. 2:**
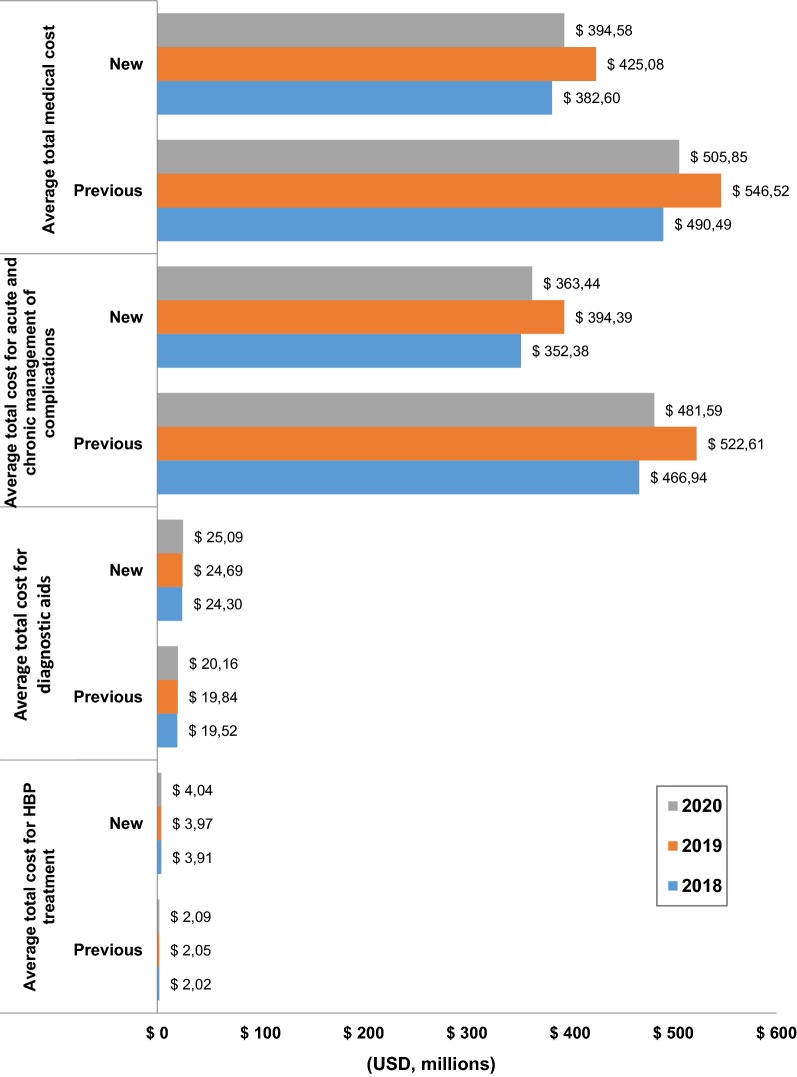
Estimated costs of antihypertensive medication, diagnostic aids and complications treatment per year, under the new scenario. Average total medical cost is equal to the sum of the direct cost of medication, diagnostic aids, and complications treatment

The model shows that lowering the cut-off points from 140/90 to 130/80 mmHg and the treatment goal (less than 120/80 mmHg) lead to a decrease in the total annual direct cost of approximately USD 108 million, which represents a reduction of more than 20% of total annual HBP costs in 2018 (see Fig. 2). Similar figures are obtained in 2019 and 2020. This decrease is mainly driven by the reduction of the acute and chronic complication management (total annual cost per episode passed from USD 466 million to USD 352 million). The opposite result was observed for medication and diagnostic aids costs which increased by 31%. Under the new hypertensive guideline, those direct costs of the HBP diagnosis and management represented approximately USD 28.2 million in 2018.

**Fig. 3:**
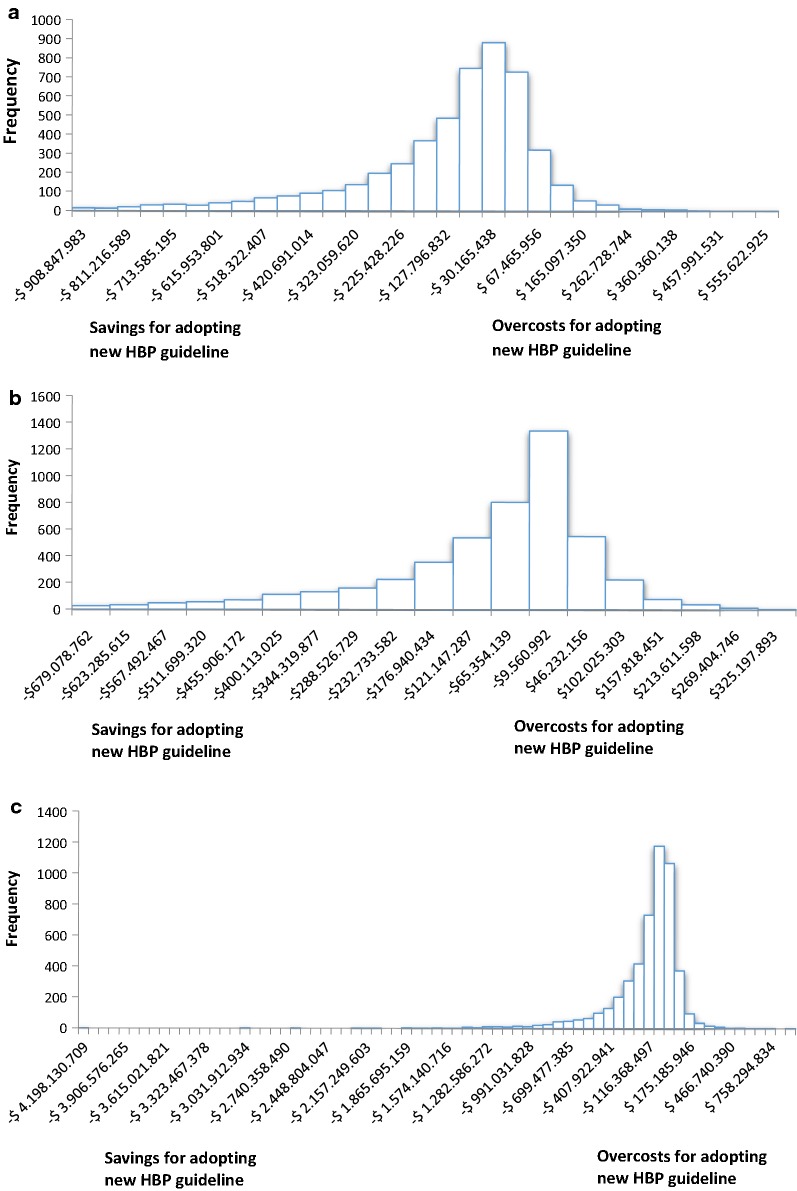
Difference of total medical costs between costs of new hypertension guideline’s adoption compared with cost of baseline scenario during the period 2018–2020. This difference was obtained from the sensitivity analysis, performed by second-order Monte Carlo simulation with 5000 iterations. **a** 2018. **b** 2019. **c** 2020

The probabilistic sensitivity analysis shows that despite of expected increase in HBP prevalence, the economic impact of the adoption of the new hypertension guideline is positive. During the period 2018–2020, 84% of the Monte Carlo simulations suggested that the new guideline represent a saving compared with baseline scenario (84.5% in 2018, 83.9% in 2019 and 84.5% in 2020). Cost saving ranged from USD 9.5 million to USD 900 million (see Fig. 3). Overcosts for adopting the new standards, were also obtained in approximately 20% of the iterations and ranged from USD 102 million to more than USD 750 million.

**Fig. 4:**
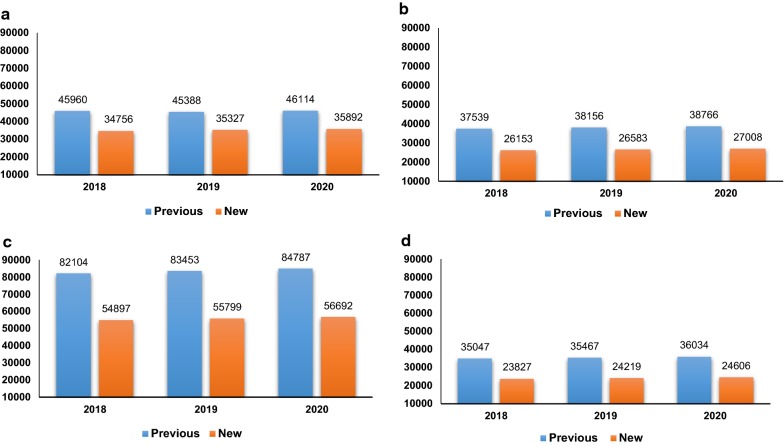
Estimated cardiovascular events according to new and baseline (previous) scenarios during the period 2018–2020. **a** Acute Myocardial Infarction. **b** Heart failure. **c** Stroke. **d** Deaths attributed to other cardiovascular causes

Figure 4 shows the estimated cardiovascular events by previous and new HBP cut-off points. Under the new cut-off points, the number of cardiovascular episodes would reduce by 22% and 33% compared with the previous guideline. The significant reductions are obtained for stroke. The estimated number of stroke using the previous guide is around 82 thousand episodes versus 55 thousand episodes adopting new HBP guideline. This result was consistent with the idea of the early diagnosis and management of hypertension might result in a reduction of cardiovascular risk events.

## Discussion

The potential budget impact model suggested that the adoption of the new HBP guideline by the AHA for diagnosis and treatment of hypertension has a high probability of saving costs in the short term for Colombian healthcare system. Under the baseline scenario, the annual direct costs of the hypertension diagnosis and management represent approximately USD 21 million (corresponds to 0.079% of health expenditure in 2014). Lowering cut-off points and target values would imply an increase of these costs by 31%, corresponding to USD 6.6 million (approximately 0.024% of health expenditure in 2014) [28]. However, the estimated costs of cardiovascular episodes decreased which translated into savings close to USD 115 million. Overall, despite the expected increase in HBP prevalence, savings would be around USD 108 million, which represents a decrease close to 22% of total annual HBP costs.

To our knowledge, there are no budget impact evaluations to compare these findings, although this “paradoxical” pattern of cost savings followed by an increase in prevalence has been described in other studies [29, 30]. Several factors may explain this pattern.

Besides the apparent reduction in costs attributed to hypertension-related complications, it is interesting the indirect role that adherence to pharmacological treatment would play. It is unlikely to attribute costs reduction to the choice of cost-effective therapies by the physician. Evidence has shown that they do not consider aspects related to costs or reimbursement measures to establishment or choice of antihypertensive treatment since their incentives are mainly in line to achieve HBP target values and reduce complications [31].

Classifying a patient as hypertensive and establishing a lower BP target value would create incentives to initiate intensive management, leading to a decrease in complications risk. Sensitivity analysis supports this finding which suggested that the level of physician and patient adherence were the primary factor to reduce costs significantly. This relationship was also described by Koçkaya et al. [32], who found that in a scenario of total adherence: the number of cases and the treatment costs could be reduced by 32%, which represented savings of USD 8.5 million and USD 72 billion respectively.

In contrast, side effects due to stricter HBP treatment goals (120/70 mmHg) might result in overmedication and hence, increase costs. SPRINT study shows that there are no significant differences in some adverse effects between the group of patients with intensive and standard treatment: 38% of patients treated targeting less than 120 mmHg exhibit serious adverse events and 37% of patients treated with a conventional target presented adverse events [11]. However, syncope, electrolyte abnormality, acute renal failure and hypotension were more common among patients in the intensive treatment group than among those in the standard treatment group [11]. Because these adverse effects are managed by changes in the therapeutic agents, changes in administration schedules and, in some cases, by reducing the dose, it was considered that they do not substantially increase costs [33]. Further research needs to compare the adverse events and the benefits associated with intensive treatment goals of HBP.

Nevertheless, some caveats of this study must be considered. The budget impact model did not consider therapeutic changes in lifestyle. It is well known that these changes contribute to a decrease in blood pressure and a lower requirement for medications [34]. Thus, it could represent an additional saving at the expense of smaller use of antihypertensive medication. Other significant complications (from the clinical and financial perspective) such as retinopathy, peripheral arterial disease, and especially hypertensive nephropathy are also not analyzed. Future research agenda should include the economic effect of adopting the new guideline on the mentioned complications.

In addition, given the limited available local data, some parameters as was mentioned above, such as increasing hypertension prevalence and the proportion of cardiovascular events were taken from AHA/ACC guideline and SPRINT-study. The population used in these studies might differ from our population, which might result in an overestimation of the budget impact of the adoption of stricter HBP treatment goals. Furthermore, subgroup analysis by sex and age were not considered in the SPRINT study, which is why it was not possible to consider the differences in cardiovascular event proportions by sex and age in the current study.

Despite of these data and methodological limitations, this study sheds the new lights of the economic impact of the recently published hypertension guideline in little-research field, estimating the potential budget impact of the new treatment standards in a middle-income country. Hypertension and complications costs represented about 1.8% of total healthcare expenditure in Colombia in 2014. Our results suggest the importance of taking care of future cardiovascular complications by adopting the recommendations of the new hypertension guideline of the AHA/ACC. It would save costs in a high-middle income country which faces a demographic trend towards population aging and significant financial sustainability difficulties.

## Conclusions

Despite the concern about the economic impact of increasing hypertension prevalence given lower high blood pressure standards, the potential budget impact related to the adoption of the new hypertension guideline is positive. Our results show that adequate and strict control of blood pressure earlier in life might decrease costs associated with complication management. This is relevant for high-middle income countries such as Colombia that exhibits high chronic prevalence and faces significant sustainability healthcare challenges at the same time. Strategies for guideline adherence by medical personnel should be reinforced to enhance the appropriate management and adequate control of blood pressure figures.

## Additional file

**Additional file 1.** Confidence intervals for estimated cardiovascular events, obtained from probabilistic sensitivity analysis.

## Abbreviations

WHO: World Health Organization
AHA: American Heart Association
ACC: American College of Cardiology
BP: blood pressure
HBP: high blood pressure
GDP: gross domestic product
AMI: acute myocardial infarction
HF: heart failure
DANE: National Administrative Department of Statistics of Colombia
SISPRO: The Integrated Information System of Social Protection of Colombia
SISMED: The Drug Price Information System of Colombia
