# Supplementary material for: Budget impact analysis of the adoption of new hypertension guidelines in Colombia
Source: Cost Eff Resour Alloc. 2018 Sep 25;16:32. doi: 10.1186/s12962-018-0152-5 (PMC6157055; doi:10.1186/s12962-018-0152-5)
Supplement: Supplementary file 1 — Additional file 1. Confidence intervals for estimated cardiovascular events, obtained from probabilistic sensitivity analysis. [file 12962_2018_152_MOESM1_ESM.pdf]

BUDGET IMPACT ANALYSIS OF THE ADOPTION OF NEW HYPERTENSION GUIDELINES IN COLOMBIA

Supplementary file 1. Confidence intervals for estimated cardiovascular events, obtained from probabilistic sensitivity analysis.

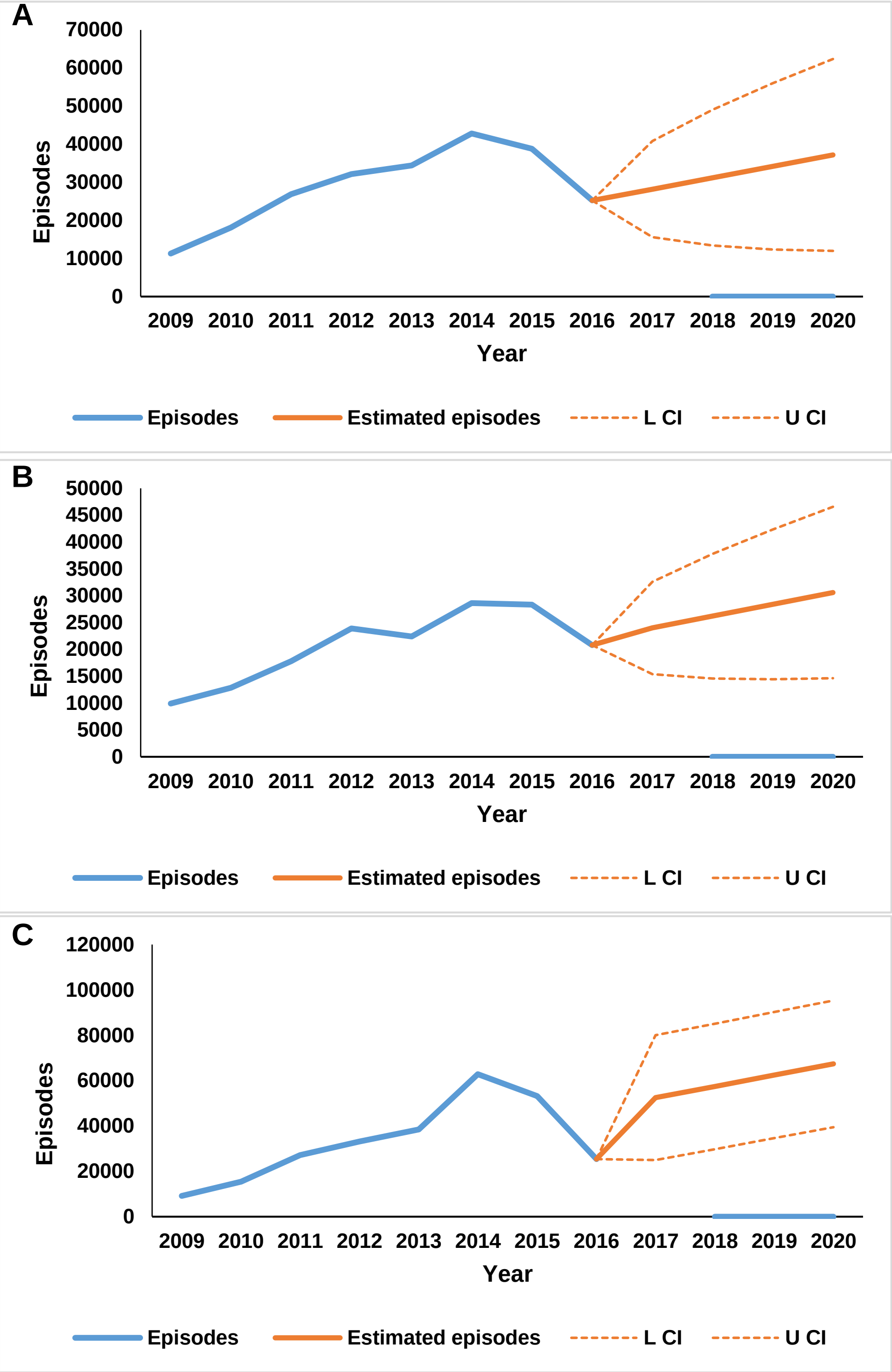

A, Acute myocardial infarction. **B,** Heart failure. **C,** Stroke. (L CI: Lower Confidence Interval. U CI: Upper Confidence Interval).
